# Efficient Derivation and Transcriptional Characterization of Mouse Extra‐Embryonic Endoderm Stem Cell Lines Generated by Somatic Cell Nuclear Transfer

**DOI:** 10.1155/sci/1968738

**Published:** 2026-06-24

**Authors:** Shuaipeng Li, Shu Wei, Guomeng Li, Mei Hu, Jiangwei Lin, Wandong Bao

**Affiliations:** ^1^ Department of Laboratory Animal Science, Kunming Medical University, Kunming, 650500, Yunnan, China, kmmc.cn; ^2^ State Key Laboratory of Genetic Evolution and Animal Models, Kunming Institute of Zoology, Chinese Academy of Sciences, Kunming, 650223, Yunnan, China, cas.cn

**Keywords:** aberrant epigenetic reprogramming, extra-embryonic endoderm stem cell line, imprinting errors, NanoString multiplex gene expression profiling, primitive endoderm, somatic cell nuclear transfer

## Abstract

Somatic cell nuclear transfer (SCNT) holds great promise for regenerative medicine and agriculture, but its application is severely hampered by low efficiency, primarily attributable to aberrant epigenetic reprogramming. Although embryonic stem cells (ESCs) and trophoblast stem cells (TSCs) have been successfully derived from cloned embryos, an in vitro counterpart of the primitive endoderm (PrE) lineage has remained unavailable. To address this gap, this study reports the first successful establishment of extra‐embryonic endoderm stem cell lines (XENs) from mouse SCNT‐derived blastocysts (NT‐XENs). Under conventional culture conditions, NT‐XENs were generated from hybrid B6D2F1 blastocysts at a high efficiency of 55%, statistically comparable to that of fertilization‐derived XEN lines (FD‐XENs, 50%), whereas derivation from inbred C57BL/6J SCNT‐derived blastocysts was markedly lower (12.5%). Immunofluorescence and NanoString multiplex gene expression profiling confirmed that NT‐XENs robustly expressed specific marker genes for PrE/XENs (e.g., *Gata4*, *Gata6*, and *Sox17*), while exhibiting negligible or absent expression of pluripotency and trophoblast markers. Based on NanoString assay data, NT‐XENs and FD‐XENs shared highly similar gene expression patterns, yet also exhibited some nonnegligible differences, exemplified by the differentially expressed genes (DEGs) *Pecam1*, *Gtl2*, *Thbd*, and *Xlr3b*. These differences raise a preliminary hypothesis that the NT‐XENs might exhibit a slight transcriptional propensity toward a more differentiated state, and potentially reflect lingering traces of SCNT‐associated epigenetic errors, such as localized dysregulation of imprinted genes and X‐linked transcripts. In summary, this study successfully establishes NT‐XEN cell lines, providing a valuable in vitro model for investigating the reprogramming scenarios of PrE lineage in SCNT and the mechanisms underlying developmental failure of cloned embryos.

## 1. Introduction

Somatic cell nuclear transfer (SCNT) is a revolutionary technology that enables the production of genetically identical individuals from differentiated somatic cells, holding immense promise for a range of applications in agriculture and regenerative medicine [1]. Despite its significant potential, the widespread application of SCNT is severely impeded by the remarkably low efficiency, that is, the vast majority of cloned embryos fail to develop to term [2]. This high rate of developmental failure is widely attributed to incomplete or erroneous epigenetic reprogramming of the donor somatic nucleus [3], in which the DNA methylation and histone modification profiles must be comprehensively remodeled into a totipotent embryonic state for achieving cloned animals [4]. Indeed, such epigenetic barriers, including defective DNA methylation of imprinted genes and abnormal transcriptomic profiles, have been widely documented during early embryonic development across various mammalian species and alternative reproductive technologies [5, 6]. Furthermore, the increasing application of advanced omics strategies has become crucial for comprehensively unraveling the molecular mechanisms underlying reproductive competence and cellular reprogramming [7]. A substantial body of evidence indicates that these reprogramming errors disproportionately compromise the development of extra‐embryonic tissues [8]. Specifically, abnormalities such as placental insufficiency and defective yolk sac formation are frequently observed in cloned conceptuses and are considered major contributing factors to their demise [9].

The formation of these critical supportive tissues is rooted in a series of precisely orchestrated cell fate decisions during early mammalian embryonic development. In the blastocyst, the first lineage segregation event establishes the trophectoderm (TE) and the inner cell mass (ICM) [10, 11]. The ICM subsequently undergoes a second lineage segregation, differentiating into the pluripotent epiblast (EPI), the source of the fetus proper, and the primitive endoderm (PrE) [12]. The PrE lineage further differentiates into two distinct layers, the parietal endoderm (PE) and the visceral endoderm (VE) [13, 14], which together construct the yolk sac indispensable for providing crucial nutritional and signaling support to the nascent embryo before the placenta becomes fully functional [15, 16]. The coordinated development and interaction of EPI, PrE, and TE are critical for embryonic viability, and their corresponding self‐renewing stem cell lines have been derived in vitro, respectively; embryonic stem cells (ESCs) from the EPI [17], XENs from the PrE [18], and trophoblast stem cells (TSCs) from the TE [19]. The availability of these stem cell lines provides a powerful and tractable toolkit for dissecting the molecular underpinnings of lineage specification and function.

Among these cellular models, XENs offer a unique *in vitro* system to study PrE‐associated biological mechanisms. They are robustly characterized by the expression of a suite of key transcription factors, such as GATA4, GATA6, SOX7, and SOX17, as well as the cell surface receptor PDGFRA [18, 20]. While both nuclear transfer‐derived ESCs (NT‐ESCs) [21, 22] and nuclear transfer‐derived TSCs (NT‐TSCs) [23] have been successfully established from SCNT‐derived embryos, offering invaluable insights into the reprogramming fidelity of the EPI and TE lineages, a stable in vitro model for the PrE lineage originating from cloned embryos has been conspicuously absent. To address this gap, our study reports the first successful derivation, with remarkable efficiency, and characterization of the XEN cell lines from SCNT‐derived blastocysts. Utilizing the targeted analysis of key developmental genes, we profiled the transcriptional state of the NT‐XENs and examined their distinctive features from those of the fertilization‐derived XEN lines (FD‐XENs).

## 2. Materials and Methods

### 2.1. Mouse Strains

The mice of wild‐type C57BL/6J and DBA/2 genetic backgrounds used in the present study were purchased from Charles River Biotechnology Co., Ltd. (Beijing, China). Mouse care and all the experimental procedures were conducted in compliance with the guidelines of the Institutional Animal Care and Use Committee (IACUC) of the Kunming Institute of Zoology, Chinese Academy of Sciences. The approval number for all contents of this research is IACUC‐RE‐2024‐01‐006.

### 2.2. Cell Culture Medium

DMEM was supplemented with 15% FBS, 1% penicillin/streptomycin, 0.1 mM nonessential amino acids, 1% β‐mercaptoethanol, 2 mM GlutaMAX supplement, 1 mM sodium pyruvate, and 1000 IU/mL leukemia inhibitory factor (LIF). This ES medium with LIF was used for deriving XENs from blastocysts.

### 2.3. Nuclear Transfer

Metaphase II (MII)‐arrested oocytes were collected from superovulated B6D2F1 females (8–10 weeks, generated by mating female C57BL/6J with male DBA/2) and cumulus cells were removed using hyaluronidase. The oocytes were enucleated in a droplet of HEPES‐CZB medium containing 5 µg/mL cytochalasin B (CB) using a blunt Piezo‐driven pipette. Then, the spindle‐free oocytes were washed extensively and maintained in CZB medium up to 2 h before nucleus injection. The cumulus cells from B6D2F1 females were gently aspirated in and out of the injection pipette to remove the cytoplasmic material and then injected into enucleated oocytes. The reconstructed oocytes were cultured in CZB medium for 1 h and then activated for 5–6 h in activation medium containing 10 mM Sr^2+^, 5 ng/mL trichostatin A (TSA) and 5 µg/mL CB. All of the reconstructed embryos were subsequently cultured in KSOM medium supplemented with 5 ng/mL TSA for another 3–4 h and then maintained in KSOM medium with amino acids at 37°C under 5% CO_2_ in air. These experimental operations followed the method of Lin et al. [24], and the method description partly reproduces their wording. For the nuclear transfer in an inbred strain background, MII‐arrested oocytes and cumulus cells from wild‐type C57BL/6J females were used, with all other experimental procedures above unchanged.

### 2.4. Derivation of XEN Cell Lines From Blastocysts

The SCNT‐derived embryos were cultured in KSOM medium with amino acids to the blastocyst stage, so were the B6D2F1 FD embryos (generated by mating female C57BL/6J with male DBA/2) after collection at the 2–8‐cell stage. Before the derivation of XEN lines, embryo quality was strictly assessed based on morphological criteria. Upon successfully reaching the expanded blastocyst stage with clearly visible ICM and TE structures, the zona pellucida was dissolved by exposure to acid Tyrode’s solution. Subsequently, individual blastocysts were plated onto 0.1% gelatin‐coated four‐well plates containing a feeder layer of mouse embryonic fibroblasts (MEFs). Culture was maintained using an ES medium containing LIF to promote XEN cell derivation, following previously established protocols [25]. The XEN lines continued to be maintained in ES medium containing LIF for passage culture once established, and the P8–P10 cells were used for subsequent molecular characterization experiments including immunofluorescence and NanoString profiling.

### 2.5. Immunofluorescence and Imaging

Immunofluorescence staining was performed as previously described [26]. Briefly, cell lines were cultured in 4‐ or 24‐well dishes. Cells were fixed in 4% paraformaldehyde at 4°C overnight or room temperature for 30 min, permeabilized with 0.1% Triton X‐100 in 1× PBS (1× PBST) for 30 min, and blocked with 5% normal donkey serum diluted in 1× PBST (blocking solution) for 1 h. Primary antibodies were diluted at 1:50–1:200 in blocking solution, and samples were then incubated at 4°C with rotation overnight. After three 10‐min washes in 1× PBST, samples were incubated in a 1:500 dilution of secondary antibody in blocking solution for 1–1.5 h at room temperature, then washed, and covered with 1× PBST containing DAPI. Images were taken with an AMG EVOS fluorescence microscope (Life Technologies). Primary antibodies from Santa Cruz Biotechnology were against Gata4 (cat. # SC‐1237), Dab2 (cat. # SC‐13982), Oct4 (cat. # SC‐5279), Nanog (cat. # SC‐376915), and Cdx2 (cat. # SC‐166830). Primary antibodies from R&D Systems were against Gata6 (cat. # AF1700) and Sox17 (cat. # AF1924). Appropriate secondary antibodies were purchased from Jackson ImmunoResearch Laboratories and Invitrogen.

### 2.6. NanoString Multiplex Gene Expression Analysis

NanoString assays were performed as previously described [26] with necessary modifications for this research. Briefly, dissociated cells were collected by trypsinization and centrifugation. Cell pellets were dispensed in RNAlater Stabilization Solution (Qiagen) and stored at −80°C for later use. Cell pellets were lysed in RLT Lysis Plus Buffer using a TissueLyser LT (Qiagen) at 40 Hz for 2 min, and then total RNA samples were extracted using RNeasy Plus Micro kit (Qiagen) according to the manufacturer’s protocol. A custom NanoString CodeSet “Extra” comprising 222 genes was designed specifically to characterize the lineage identity and reprogramming errors in the NT‐XEN lines derived in this study. The selection criteria for these genes included (1) core markers for pluripotency and specific early embryonic lineages (EPI/ESCs, PrE/XENs, and TE/TSCs) and (2) epigenetic regulators and imprinted genes susceptible to reprogramming errors during SCNT. For each total RNA sample, an aliquot of 100 ng was hybridized at 65°C for 18 h and processed with a nCounter Analysis System GEN1 (NanoString Technologies).

Raw reporter counts (Additional file 1: Table S1) were processed using nSolver Analysis Software (NanoString) in a stepwise manner. First, standard quality control checks were performed to ensure data integrity regarding imaging, binding density, and positive control linearity. Second, background subtraction was conducted based on the built‐in negative controls (Additional file 1: Table S2). Third, positive control probes were used to assess the assay performance and technical consistency across samples (Additional file 1: Table S3). Finally, the housekeeping normalization factors calculated from the geometric mean of commonly used housekeeping genes Actb and Gapdh (Additional file 1: Table S4) were used for the final normalization of expression counts (Additional file 1: Table S5). The normalized counts were used for differentially expressed gene (DEG) analysis and log_2_‐transformed for correlation clustering analysis and heatmap visualization.

### 2.7. Statistical Analysis

The derivation efficiencies of XEN lines were compared using a two‐sided Fisher’s exact test in GraphPad Prism v9.0. For NanoString data, inter‐sample correlation was evaluated using the Pearson correlation coefficient. DEGs between NT‐XENs and FD‐XENs were identified using the limma package in R (fold change >2, *p*  < 0.05), based on empirical Bayes methods. All experiments were conducted with independent biological replicates.

### 2.8. Figure Preparation

**Figure 1:**
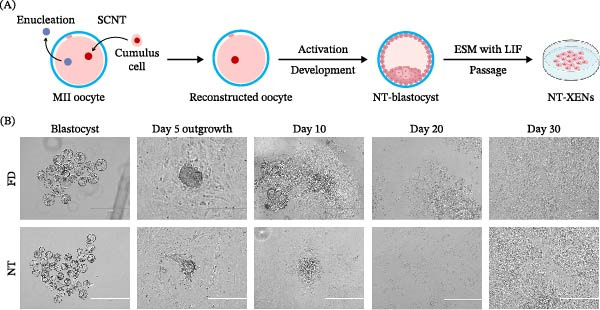
Derivation of XENs from mouse SCNT‐derived blastocysts. (A) Experimental strategy for establishing mouse nuclear transfer‐derived XEN cell lines. MII oocytes were enucleated and received nuclear transfer from cumulus cells to form reconstructed oocytes, which were subsequently activated and developed to the blastocyst stage in vitro. These SCNT‐derived blastocysts were then cultured in ES medium with LIF to derive XEN cells, and the NT‐XEN cell lines were ultimately established via continuous subculture. ESM, ES medium; LIF, leukemia inhibitory factor; MII, metaphase II; SCNT, somatic cell nuclear transfer. (B) The process of deriving XEN cells, respectively from mouse fertilization‐derived and SCNT‐derived blastocysts, and establishing their cell lines in vitro under bright‐field microscopy. The numbers of days cultured in ES medium with LIF are indicated. Scale bar: 400 μm.

**Figure 2:**
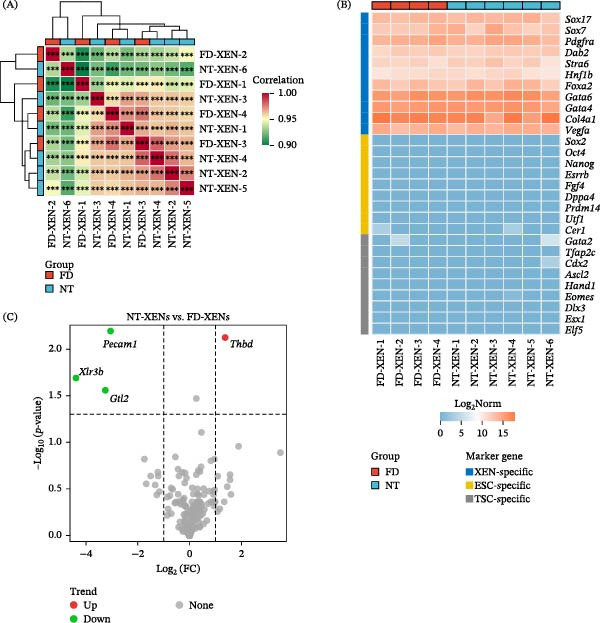
Characterization of gene expression profiles of mouse NT‐XENs using NanoString. (A) Hierarchical clustering analysis of correlation among all NanoString‐assayed samples (including four independent FD‐XEN cell lines and six independent NT‐XEN cell lines). Correlation values were calculated based on the log_2_‐transformed normalized counts.  ^∗∗∗^ *p* < 0.001. (B) Expression abundance of XEN‐, ESC‐, and TSC‐specific marker genes across all NanoString‐assayed samples. The heatmap was plotted using the log_2_‐transformed normalized counts. ESC, embryonic stem cell; TSC, trophoblast stem cell. (C) The volcano plot presents the DEG analysis result between NT‐XENs and FD‐XENs based on the NanoString assay data. Fold change (FC) >2, *p*‐value <0.05.

The experimental workflow diagram shown in Figure 1A was created using the graphical assets from BioGDP.com [27]. Multichannel‐merged immunofluorescence images were created using ImageJ. All NanoString assay data‐associated figures in Figure 2 were plotted using the Metware Cloud (https://cloud.metware.cn).

## 3. Results

### 3.1. Efficient Derivation of XEN Cell Lines From SCNT‐Derived Blastocysts

To derive NT‐XEN cell lines, we reconstructed embryos by enucleating the MII oocytes from hybrid B6D2F1 females (generated by mating female C57BL/6J with male DBA/2) and injecting the nuclei from their own cumulus cells. The reconstructed embryos were cultured to the blastocyst stage in vitro and then used to establish XEN lines in ES medium supplemented with LIF (Figure 1A). As a control, FD blastocysts of B6D2F1 background were also processed under identical culture conditions for XEN line derivation after their in vitro development from the early cleavage stage.

Specifically, we cultured the reconstructed embryos in KSOM medium until the blastocyst stage and then removed the zona pellucida. A random selection of 20 SCNT‐derived blastocysts were separately seeded into the wells coated with 0.1% gelatin and covered with MEFs and switched to ES medium with LIF. After 3 days in culture, the blastocysts began to form outgrowths, which were subsequently disaggregated on Day 5. With extended culture, stable XEN cell lines were ultimately established around Day 30 (Figure 1B). We thus derived, using the conventional method with ES medium and LIF [25], a total of 11 XEN cell lines from 20 SCNT‐derived blastocysts at an efficiency of 55% (Table 1). For the FD group, a very similar process of establishing XEN cell lines was observed under the same experimental conditions, and 10 XEN cell lines were finally derived from 20 FD blastocysts at an efficiency of 50% (Table 1). Thus, it could be concluded that NT‐XENs showed a derivation efficiency from blastocysts as high as that of FD‐XENs without statistically significant difference (*p* = 1.0, Fisher’s exact test).

**Table 1:** The establishment efficiency of XEN cell lines from fertilization‐derived (FD) and SCNT‐derived (NT) blastocysts in this study.

Deriving type	Culture medium	Genetic background	No. of blastocysts	No. of outgrowths	No. of XEN cell lines (%)
FD	ES medium with LIF	B6D2F1	20	20	10 (50)^ns^
NT	ES medium with LIF	B6D2F1	20	13	11 (55)^ns^, ^∗∗^
NT	ES medium with LIF	C57BL/6J	40	26	5 (12.5) ^∗∗^

*Note:* Statistical significance of the efficiency difference between groups was determined using a two‐tailed Fisher’s exact test. ns, not significant, between the FD and NT groups in the B6D2F1 background (*p* = 1.0);  ^∗∗^ *p* < 0.01, between the NT groups in the B6D2F1 and C57BL/6J background, respectively (*p* = 0.0012).

**Figure 3:**
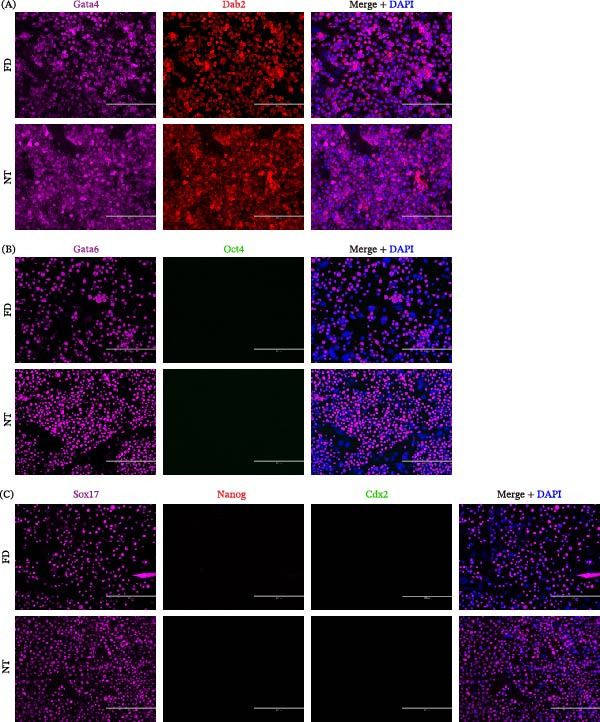
Immunofluorescence staining validates the marker protein expressions in mouse NT‐XENs. The XEN‐specific markers, including Gata4 and Dab2 (A), Gata6 (B), and Sox17 (C) were strongly detected in mouse NT‐XENs, whereas the ESC‐specific markers Oct4 (B) and Nanog (C), and the TSC‐specific marker Cdx2 (C) showed no detectable signal. Scale bar: 200 μm.

Using immunofluorescence staining, we detected the expression of stem cell‐specific markers in the derived NT‐XENs. Like FD‐XENs, the NT‐XENs were able to significantly express the specific markers Gata4, Dab2, Gata6, and Sox17, but were negative for the ESC‐specific markers Oct4 and Nanog, and negative for the TSC‐specific marker Cdx2 (Figure 3A–C), thereby confirming their identity as XEN cell lines.

### 3.2. NanoString Assays Validated the Highly Similar Gene Expression Patterns in NT‐ and FD‐XENs

Furthermore, we applied the NanoString multiplex platform for detecting more information on the gene expression profiles of FD‐XENs and NT‐XENs, respectively. A total of 222 genes, including the numerous genes with enriched expression in the early embryonic cell lineage‐derived stem cell lines (e.g. ESCs, XENs, and TSCs), were detected in four FD‐XEN lines and 6 NT‐XEN lines (Additional file 1: Tables S1–S5). Inter‐sample correlation clustering analysis showed that the FD‐XEN and NT‐XEN line samples were highly intermingled in clustering (rather than separated by group), with significant positive correlations between the replicates of the two groups (*p*  < 0.001, Figure 2A), indicating that the NT‐XEN lines should share a highly similar gene expression pattern with the FD‐XEN lines based on the targeted marker genes. Of particular focus was that, similar to the FD‐XEN lines, all of the NT‐XEN lines presented a high abundance expression of the known XEN‐specific genes (e.g. *Sox17*, *Sox7*, *Pdgfra*, *Gata6*, *Gata4*, and *Dab2*) versus low or no expression of ESC‐specific genes (e.g. *Sox2*, *Oct4*, *Nanog*, *Esrrb*, and *Prdm14*) and TSC‐specific genes (e.g., *Gata2*, *Cdx2*, *Elf5*, and *Tfap2c*) (Figure 2B), which thus confirmed and extended the immunofluorescence profiles. All these genetic expression characteristics further verified our high‐quality derivation of XENs from SCNT‐derived blastocysts.

### 3.3. Minimal Targeted Transcriptional Divergence Between FD‐XENs and NT‐XENs

Based on the NanoString assay data, we performed DEG analysis between FD‐XENs and NT‐XENs. As expected, the 222 surveyed genes showed virtually no differential expression overall; nevertheless, the four genes, *Pecam1*, *Thbd*, *Xlr3b*, and *Gtl2*, exhibited differential expression trends between the two XEN types, with *Thbd* upregulated and the other three downregulated in NT‐XENs (fold change > 2, *p*‐value < 0.05, Figure 2C, Additional file 1: Table S6). *Pecam1* is a pluripotency marker gene that is restrictedly expressed in the ICM and then maintained in EPI, but not in PrE, during the second cell fate determination in blastocysts [28]. As XEN lines are the in vitro counterparts of PrE [25], the downregulated *Pecam1* expression here might suggest a more differentiated transcriptional state for the NT‐XENs compared with the FD‐XENs. Potentially in accordance with this, another pluripotency marker gene *Gtl2*, a key maternal‐allele‐expressed long noncoding RNA (lncRNA) gene located in the *Dlk1*–*Dio3* imprinted domain essential for pluripotency regulation [29] and identified to exhibit restricted expression in ICM [30], was also further down‐regulated in the NT‐XENs. Moreover, *Thbd*, a gene preferentially expressed in the former of PE and VE [31, 32] that both derive from PrE in vivo, was upregulated in the NT‐XENs compared with the FD‐XENs, which leads to a preliminary hypothesis that NT‐XENs might possess a transcriptional propensity slightly towards the PE direction, though this warrants future functional validation. *Xlr3b* is also a maternal‐allele‐expressed imprinted gene, which is X‐linked and subject to X‐chromosome inactivation (XCI) [33]. The downregulation of *Xlr3b* expression in the NT‐XENs could be possibly explained by the aberrant XCI effect that nonrandomly appears in the SCNT embryos [34] and is speculated to exist in their derivations, including the NT‐XENs. To summarize, the NanoString assay data revealed that the NT‐XENs shared a highly similar marker gene expression profile with the FD‐XENs but still possessed minimal yet nonnegligible transcriptional divergence from the latter.

### 3.4. Lowered Derivation Efficiency of NT‐XEN Lines With Inbred Genetic Background

Besides, we also utilized the MII oocytes and cumulus cells from C57BL/6J females to obtain reconstructed embryos, which were then cultured and seeded for deriving XEN lines under identical experimental conditions. Eventually, we obtained only five XEN cell lines from 40 C57BL/6J SCNT‐derived blastocysts, at an efficiency of 12.5% that was significantly lower than that of the B6D2F1 NT‐XEN line derivation (55%; *p*  < 0.01, Fisher’s exact test; Table 1). This indicated a serious impact of the inbred genetic background on the derivation efficiency of XEN lines from SCNT‐derived blastocysts when compared with that of XEN line derivation from the SCNT‐derived blastocysts with a hybrid genetic background.

## 4. Discussion

In the present study, we report the first derivation of stable XEN cell lines from SCNT‐derived blastocysts, providing a utilizable and renewable in vitro resource for investigating the reprogramming fidelity of the PrE lineage following SCNT. It is crucial, however, to acknowledge that cell lines derived in vitro may not perfectly represent the state of their in vivo counterparts [35]. The derivation process itself imposes a strong selective pressure, meaning our NT‐XENs represent the outcome of both nuclear reprogramming and subsequent in vitro adaptation. Nevertheless, NT‐XENs should be invaluable for their ability to capture and highlight the stable molecular errors in reprogramming, as studies on NT‐ESCs have consistently shown that certain epigenetic aberrations, such as those affecting imprinted genes or XCI, are tenaciously retained through extensive passaging [36].

A notable finding was the high derivation efficiency of NT‐XENs from hybrid B6D2F1 blastocysts (55%), which was comparable to that of FD controls (50%) without a statistically significant difference. This is consistent with the reports on both NT‐ESCs [22] and NT‐TSCs [23], where derivation efficiencies can also rival those of conventional counterparts under optimized conditions. It should be noted that this high efficiency does not necessarily imply flawless reprogramming within the PrE of SCNT‐derived blastocysts. A more plausible interpretation is that SCNT accomplishes a sufficient degree of initial reprogramming to activate the core regulatory network for PrE formation, and the subsequent stringent in vitro culture conditions select for the most successfully reprogrammed cells to proliferate and form stable lines. In accordance with the latter, the derived NT‐XENs showed highly similar transcriptional profiles with the FD‐XENs based on the NanoString assay data. Thus, the high efficiency here likely reflects a combination of adequate initial reprogramming and stressful in vitro selection.

In stark contrast to the B6D2F1 hybrid, the derivation efficiency of NT‐XEN lines from the inbred C57BL/6J background was drastically reduced (12.5%). This strain‐dependent developmental bottleneck has long been documented phenotypically in both cloning efficiency [37] and the establishment of NT stem cell lines, for example, NT‐ESCs [38]. Mechanistically, this discrepancy likely stems from the lack of genetic diversity in the inbred strain, which may result in weaker allelic complementation and functional tolerance to buffer the impact of SCNT‐associated epigenetic aberrations compared with the hybrid vigor in heterozygous backgrounds. Consequently, the inbred cloned embryos tend to exhibit extremely restricted developmental competence, thereby limiting the successful derivation of NT‐XEN lines in the C57BL/6J background.

Despite the overall transcriptional similarity between NT‐XENs and FD‐XENs based on the NanoString profiling data, the few DEGs we identified may predict key molecular signatures of incomplete reprogramming. The concurrent downregulation of pluripotency‐associated genes (*Pecam1* and *Gtl2*) [28, 30] and upregulation of a PE‐preferential gene (*Thbd*) [39] suggest that NT‐XENs might exhibit a more differentiated or lineage‐restricted transcriptional propensity. This seems to align with reports that cloned embryos can exhibit tendencies toward premature differentiation [40]. The downregulation of *Gtl2*, a critical component of the *Dlk1*‐*Dio3* imprinted domain essential for high‐quality pluripotency [29, 41], further reinforces this notion. Besides, the downregulation of the X‐linked imprinted gene *Xlr3b* is highly consistent with the aberrant XCI and subsequent misexpression of X‐linked genes frequently documented in SCNT embryos [34, 42]. Although these targeted transcriptional changes merely provide preliminary molecular hints rather than definitive proof of global epigenetic failure, they point to the utility of NT‐XENs as a model for studying such defects.

Finally, several limitations exist in the present study. For example, the characterization of NT‐XENs was primarily restricted to targeted transcriptomic profiling (NanoString) and immunofluorescence. While sufficient for confirming cellular identity, targeted panels cannot replace genome‐wide or allele‐specific RNA‐seq, which is able to help comprehensively evaluate global reprogramming fidelity or imprinting status. Also, our hypothesis regarding the potential more differentiated or lineage‐biased (PE direction) propensity of NT‐XENs is based simply on the dysregulation of limited transcriptional markers. Future studies employing rigorous functional evaluations, such as in vitro directed differentiation assays and in vivo chimera contribution experiments, are essential to definitively determine the functional characteristics of NT‐XENs compared with their FD counterparts. Therefore, the findings presented here should serve as a valuable initial establishment of the cellular model and a preliminary transcriptional screening, paving the way for future in‐depth omics and functional investigations on this special XEN type.

## 5. Conclusions

Our study successfully establishes and characterizes NT‐XEN cell lines, demonstrating that while their cellular identity and targeted marker expressions are remarkably similar to their FD counterparts, they harbor detectable transcriptional differences that may potentially reflect the molecular legacy of their SCNT origin. The establishment of these cell lines fills a critical gap in the field, providing an important and previously unavailable resource for exploring the molecular signatures causally linked to the yolk sac abnormalities and developmental failure endemic to cloned embryos.

## Author Contributions

Wandong Bao and Jiangwei Lin conceived the study, drew the diagrams, and drafted the manuscript. Shuaipeng Li and Shu Wei designed the experiments. Shuaipeng Li, Shu Wei, Guomeng Li, and Mei Hu performed the experiments.

## Funding

This work was supported by the National Natural Science Foundation of China (Grants 31970823 and 32270862).

## Disclosure

All authors have read and approved the final version of the manuscript. This manuscript was previously posted as a preprint on bioRxiv (https://www.biorxiv.org/content/10.64898/2026.02.22.707260v1) [43].

## Conflicts of Interest

The authors declare no conflicts of interest.

## Supporting Information

Additional supporting information can be found online in the Supporting Information section.

## Supporting information


**Supporting Information** Additional file 1: NanoString data processing and differential expression analysis results (Table S1–S6). Table S1: Raw NanoString counts of FD‐XEN and NT‐XEN cell lines. Table S2: Background‐subtracted raw NanoString counts of FD‐XEN and NT‐XEN samples. Table S3: Quality control metrics of positive controls for NanoString analysis. Table S4: Housekeeping gene normalization metrics for NanoString analysis. Table S5: Normalized NanoString counts of FD‐XEN and NT‐XEN samples. Table S6: DEG analysis result of NT‐XENs vs. FD‐XENs based on the NanoString assay data.

## Data Availability

All data generated or analyzed during this study are included in this article and its supporting information files. The processed and analyzed NanoString gene expression data are available in Additional file 1.
